# Direct numerical simulation of an unsteady wall-bounded turbulent flow configuration for the assessment of large-eddy simulation models

**DOI:** 10.1038/s41598-023-37740-7

**Published:** 2023-07-11

**Authors:** Linus Engelmann, Josef Hasslberger, Seung-Jin Baik, Markus Klein, Andreas Kempf

**Affiliations:** 1 Fluid Dynamics, Institute for Energy and Materials Processes (EMPI), University of Duisburg-Essen, Carl-Benz-Strasse 199, 47057 Duisburg, Germany; 2 Department of Aerospace Engineering, Institute of Applied Mathematics and Scientific Computing, University of the Bundeswehr Munich, Werner-Heisenberg-Weg 39, 85577 Neubiberg, Germany

**Keywords:** Aerospace engineering, Applied mathematics, Mechanical engineering

## Abstract

A new benchmark case for the evaluation of direct numerical simulation (*DNS*) and large-eddy simulation (*LES*) models and methods is presented in this study. The known Taylor–Green vortex is modified by replacing the periodic boundary conditions in one direction with a no-slip boundary. A passive scalar is added and transported from the wall into the fluid. The addition of walls allows for the study of transient-instationary flows in a simple geometry with clean boundary and initial conditions, which is a key requirement for the assessment of LES modeling strategies. The added scalar mimics heat transfer through the wall. The case features reasonable computational cost for highly-resolved LES and DNS calculations. Simulations of the wall-bounded Taylor–Green vortex are easy to setup and do not require additional modeling. The proposed modification of the case is compared to the default Taylor–Green vortex and the difference in flow-physics is discussed. A detailed convergence study with four meshes, each of them refined by a factor of 2, has been conducted. The results reveal that converged second-order statistics can be obtained up to a dimensionless time of $t/t_0 = 20$. Beyond that, due to the unsteady chaotic nature of the flow, some uncertainties remain. The results show that the case features challenging (near-wall) flow dynamics, which cannot be covered using the default Taylor–Green vortex and hence, justify the proposed case as a useful benchmark.

## Introduction

Turbulent boundary layers are ubiquitous in many engineering and environmental applications, and the study of unsteady turbulent boundary layers is of paramount importance in many fields, ranging from aerodynamics and hydrodynamics to chemical and biological processes. Understanding the dynamics of unsteady turbulent boundary layers can provide valuable insights into fundamental aspects of turbulence and improve the design and performance of practical systems. Despite the computational and modeling challenges, significant progress has been made in recent years in the field of numerical simulations of unsteady turbulent boundary layers^1–3^. One important reason for studying unsteady turbulent boundary layers is their presence in many practical flows that exhibit unsteady behavior, such as the flow around aircraft wings, wind turbines, and ocean currents. Another reason is to gain insights into fundamental aspects of turbulence. Unsteady and oscillating turbulent boundary layers have also been studied extensively in various contexts. For example, in lakes and coastal oceans, oscillating turbulent boundary layers caused by surface and internal waves play a crucial role in mixing and transport processes^4^. In piston engines, the non-stationary nature of the boundary layers affects the heat and mass transfer, and accurate predictions of the flow behavior are crucial for device optimization^5–7^. In biomedical applications such as blood flow analysis, the study of unsteady and oscillating turbulent boundary layers is important for understanding and predicting wall shear stress and total pressure in vessels and veins^8^. In contrast to oscillating laminar boundary layers (such as Stokes second problem which has an exact solution in the laminar regime^5^) or steady equilibrium boundary layers, there are no analytical models for oscillating turbulent bottom boundary layers and direct measurement of the kinematics remains difficult^9^. In oscillating boundary layers it can happen that the pressure gradient term in the momentum equation is not anymore balanced by the Reynolds stress, but by the rate of change term. This can give rise to deviations from the well-known log-law behavior^9^. Such information is very important for resource efficient wall modeling. Spalart and Baldwin^10^ study the turbulent boundary layer under a free stream velocity that varies sinusoidally in time in the context of Reynolds averaged Navier–Stokes (*RANS*) modeling. The accuracy of several closure models of the RANS equations in predicting the characteristics of an oscillating turbulent wall boundary layer has been analyzed by Cavallaro et al.^11^. Radhakrishnan and Piomelli^12^ perform large-eddy simulation (*LES*) of oscillating boundary layers and compare different subgrid-scale (*SGS*) models as well as different treatments of the wall layer. It is found that models that are excessively dissipative do not respond quickly enough to the changes caused by the free stream.

The wall-bounded Taylor–Green vortex (*WTGV*) studied in this work represents a very simple and well-defined flow configuration with very rich physics featuring laminar-turbulent transition in the bulk flow combined with unsteady boundary layer dynamics caused by the evolving Taylor–Green vortex (*TGV*) flow structures. Hence, it is believed that the WTGV potentially represents a very useful benchmark test case for the future development of turbulence models for complex unsteady wall bounded flows. The initialization of the configuration is trivial because the initial flow field is represented by a single wavelength which corresponds to the dimensions of the computational box. Hence, filtering DNS data to obtain LES initial conditions is obsolete. This applies for all possible Reynolds numbers. Due to its relatively short computational run-time, high resolution can be achieved at low computational cost. Moreover, these advantages make the case an excellent choice for in-the-loop machine learning studies, which has already been utilized for the default TGV configuration by Reissmann et al.^13^.

## Case description

Laminar-turbulent transition forms a highly challenging case for LES models. The understanding and prediction of the transition is crucial in numerous application-fields such as wing aerodynamics, reactor-design or marine-engineering. Most of the applications involving laminar-turbulent transition feature boundary layers due to the presence of walls. A prominent benchmark for the study of free laminar-turbulent transition is the TGV. To increase the relevancy of this case, the concept of transition and decaying turbulence is combined with the concept of wall-driven turbulence. Thus, a temporally evolving vortex system is chosen which is bounded by two parallel walls.

The case-design is based on the well-known Taylor–Green vortex configuration^14,15^. The domain features a length of $2\pi$ in each dimension. The initial velocity field is given by the following expression:

1 $$\begin{aligned} \begin{aligned} u\left( x,y,z,t=0\right)&= U_0\cos \left( x\right) \sin \left( y\right) \sin \left( z\right) \\ v\left( x,y,z,t=0\right)&= 0\\ w\left( x,y,z,t=0\right)&= -U_0\sin \left( x\right) \sin \left( y\right) \cos \left( z\right) \end{aligned} \end{aligned}$$

with the reference velocity $U_0 = 1\ \text {m/s}$, the reference timescale $t_0 = 1\ \text {s}$ and the reference length $l_0 = 1\ \text {m}$. To make the setup suitable for the study of wall-driven turbulence, two parallel walls are added normal to the *y*-direction, consistent with the fact that the initial values of *v* are set to zero. The *u* and *w* velocity component are also zero at the wall because of the factor $\sin \left( y\right)$ according to Eq. 1. The Reynolds number of $\text {Re} = \rho U_0 l_0 / \mu = 1600$ at the initialization is achieved by setting the density to $\rho = 1 \text {kg/m}^3$ and the dynamic viscosity is set to $\mu = 0.625 \cdot 10^{-3} \text {Pa s}$.

The scalar $\phi$ is initialized with values of zero throughout the domain. The cell-faces at the wall are forced to hold constant values:

2 $$\begin{aligned} \begin{aligned} \phi \left( x,y=0,z,t\right)&= 1 \\ \phi \left( x,y=2\pi ,z,t\right)&= 1 \end{aligned} \end{aligned}$$

And the scalar values at the fluid-wall interface do not change with time. This allows for the observation of wall-to-fluid scalar-transfer by convection and diffusion.

## Numerical methods

All calculations are performed using the in-house solver *PsiPhi*, which has been developed at the Imperial College London and the University of Duisburg-Essen. The solver applies a finite-volume method (*FVM*) on a cubic equidistant grid. *PsiPhi* has been used in earlier application- and model-development orientated studies^16–18^. In this study the incompressibly-formulated Navier–Stokes equations of momentum and a passive scalar are solved. The equations read:

3 $$\begin{aligned} \begin{aligned} \frac{\partial \rho }{\partial t} + \frac{\partial {\rho u_{\text {j}}}}{\partial x_{\text {j}}}&= 0 \\ \frac{\partial \rho {u_i}}{\partial t} + \frac{\partial \rho {u_{\text {i}}} {u_{\text {j}}}}{\partial x_{\text {j}}}&= -\frac{\partial {p}}{\partial x_{\text {i}}} + \frac{\partial }{\partial x_{\text {j}}} \left( \mu \left( \frac{\partial {u_{\text {i}}}}{\partial x_{\text {j}}} + \frac{\partial {u_{\text {j}}}}{\partial x_{\text {i}}} \right) -\frac{2}{3}\mu \frac{\partial u_{\text{k}}}{\partial x_{\text{k}}} \delta_{\text{ij}} \right) \\ \frac{\partial \rho {\phi }}{\partial t} + \frac{\partial \rho {\phi } {u_{\text {j}}}}{\partial x_{\text {j}}}&= \frac{\partial }{\partial x_{\text {j}}} \left( D_{\phi } \left( \frac{\partial \rho {\phi }}{\partial x_{\text {j}}}\right) \right) \end{aligned} \end{aligned}$$

The momentum transport equations are spatially discretized using fourth-order central differencing combined with the midpoint rule of integration for the DNS calculations. The additional scalar transport equation is also discretized using fourth-order central differencing. The solution is advanced in time with an explicit low-storage third-order Runge–Kutta scheme using a CFL number of 0.3. The Poisson-equation is solved using a Jacobi solver. *PsiPhi* uses a distributed memory, domain decomposition approach for parallelization, utilizing MPI (*Message Passing Interface*) communication, the number of cells and cores can be found in Table 1.

**Table 1:** Computational cost for the wall-bounded Taylor–Green vortex DNS.

Cells/dimension [#]	Cells/total [#mio]	Runtime [CPUh]
256	16.8	141.23
512	134.2	2259.62
1024	1073.7	36,154.03
2048	8589.9	578,464.23

## Results

Direct Numerical Simulation of the TGV and the WTGV were performed at different grid-resolution levels to establish grid-convergence on the finest grid. While the required DNS-resolution for the TGV is known due to studies by e.g. Brachet et al.^14,15^, the consideration of walls adds turbulence production at the walls to the system, which increases the required resolution. To maintain the requirement of the first wall-adjacent cell lying within the first viscous wall-unit – typically more cells are recommended –, grid-resolutions of up to $2048^3$ cells were investigated. The resolution achieved by the grid changes with time due to the non-stationary nature of the flow and the least number of cells in the first viscous wall unit were found in the interval of $t/t_0$ from 5 to 10, leaving at least two cells within $y^+ < 1$. It was found that the calculations show grid-convergence and a grid of $512^3$ cells offers good resolution for both TGV and WTGV, showing convergence for the volume averaged flow statistics.

### Flow structure

**Figure 1:**
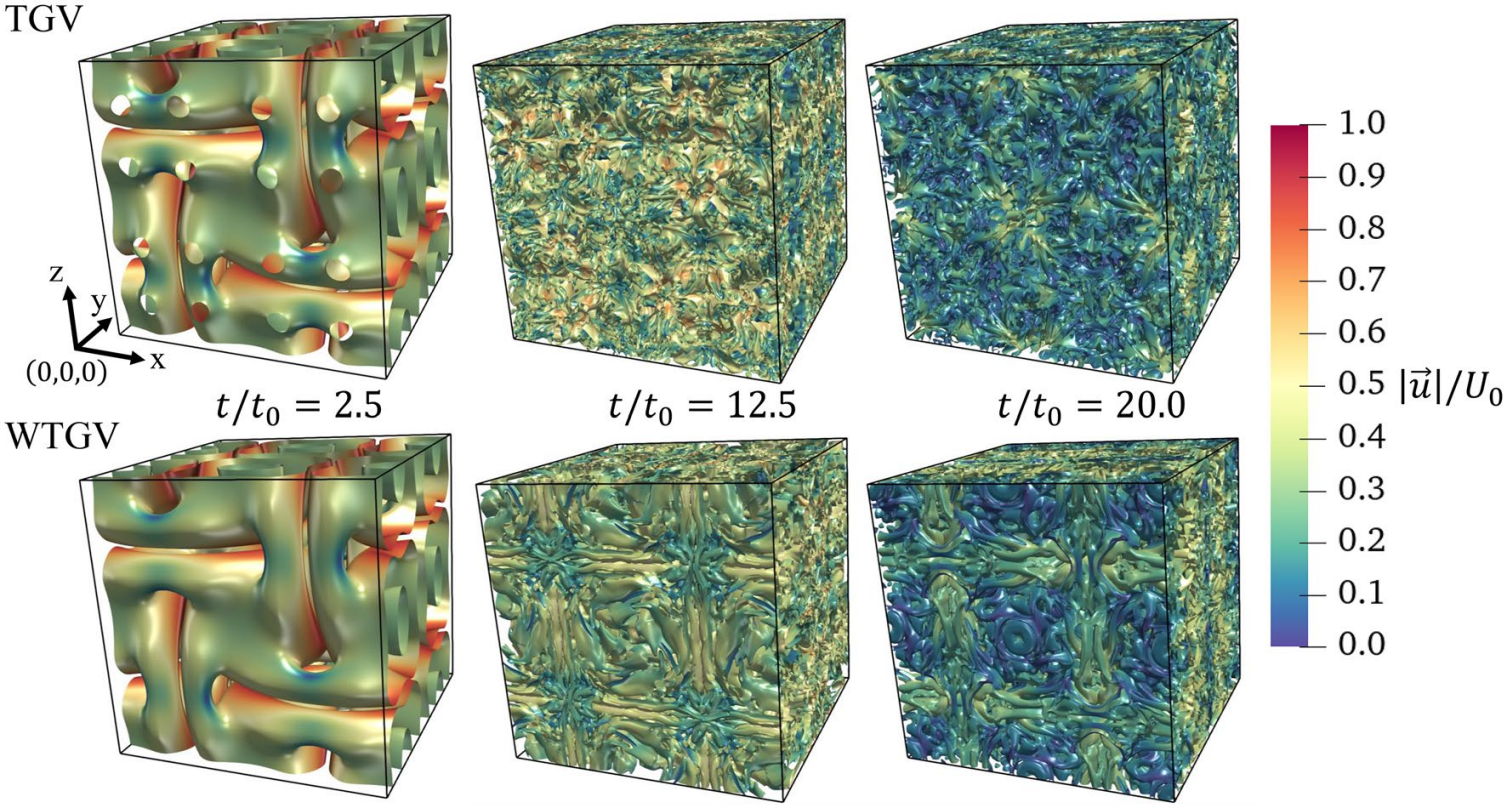
Q-criterion iso-surfaces at a value of zero colored using the velocity magnitude at dimensionless times of $t/t_0 = 2.5$, $t/t_0 = 12.5$ and $t/t_0 = 20.0$ on the grid of $256^3$ cells. The upper row is the default TGV, the lower row shows the WTGV - the front face is the wall-adjacent side.

The flow structure of the TGV and the WTGV is illustrated by the Q-criterion iso-surfaces at a value of zero at three different timesteps in Fig. 1. The iso-surfaces are colored by the velocity magnitude. The similarities of the free and wall-bounded TGV are more pronounced at early times due to the same initialization. The iso-surface of the wall-bounded case appears continuous, while the default case surface is penetrated by non-zero circular holes in the $y=0$ plane center. The later timesteps show more disordered structures for the default case. The wall-bounded case reveals a distinct torus-shaped structure in the center of the plane. This structure is rather subtle at dimensionless times of 12.5 but more pronounced at 20.0. The coloring of the iso-surfaces reveals similar velocity levels at the first two images. For the WTGV at time 20.0, however the low velocity regions are more distinct due to the no-slip condition at the wall.

### Globally averaged statistics

**Figure 2:**
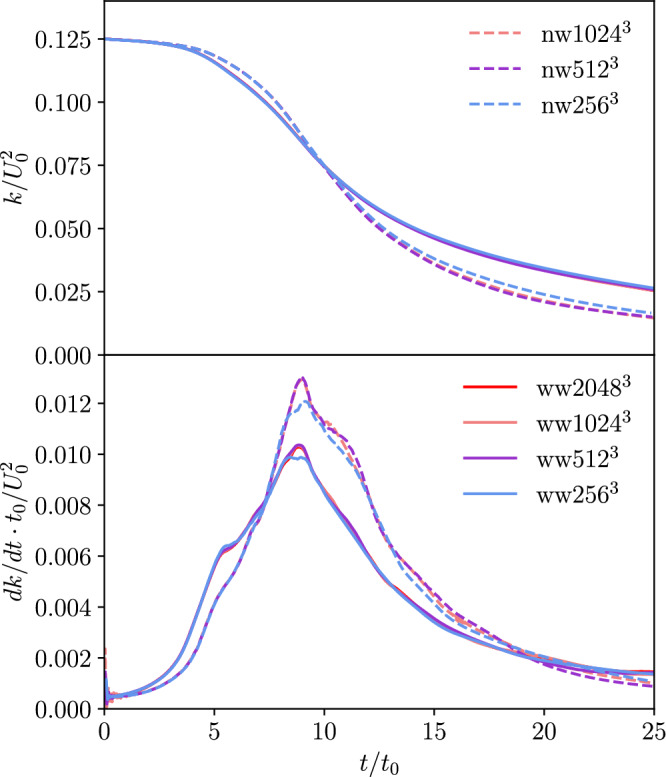
Evolution of the normalized volume-averaged kinetic energy *k* and the dissipation rate *dk*/*dt* with time for all grid-resolutions of the TGV (*nw*) and WTGV (*ww*).

Figure 2 shows the normalized volume-averaged kinetic energy $k/U^2_0$ and its dissipation rate $dk/dt \cdot t_0/U^2_0$ profiles with time for the TGV and WTGV case. In the plots the TGV results will be indicated as no-wall (*nw*) and the WTGV results as with-wall (*ww*). The curves are presented for the different grid resolutions. Little differences can be observed for the kinetic energy profile for the different grids. Slight differences can be observed in the time interval of 10–15 which remain till the end of the simulation without growing further. More differences can be found for the dissipation rate profile. While there are no differences up to the dimensionless time 8, the grid of $256^3$ cells does not fully capture the peak as observed in the two finer grids. Negligible differences occur for the later simulation times.

**Figure 3:**
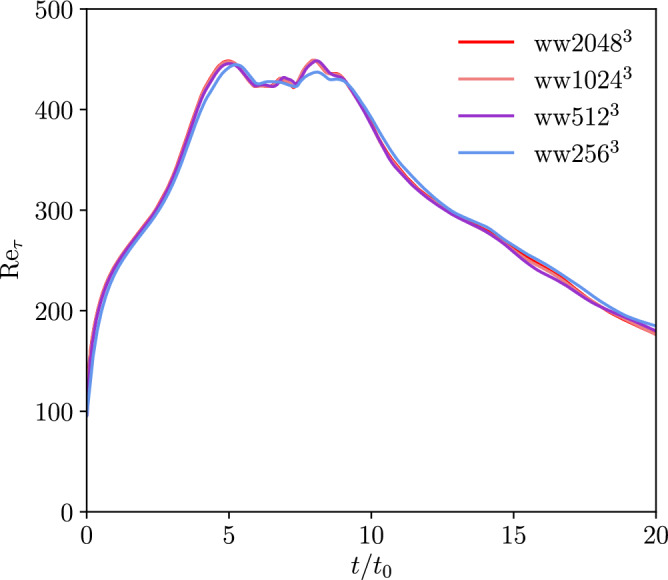
Evolution of friction Reynolds number Re$_{\tau }$ with time for all grid-resolutions of the WTGV.

In Fig. 3, the friction velocity based Reynolds number Re$_{\tau } = u_\tau L_0/\mu$ can be seen for all grid-resolutions. This Reynolds number is a measure for the velocity gradient at the wall and varies over time due to laminar-turbulent transition including vortex breakdown. It is calculated based on the friction velocity $u_\tau = \sqrt{\tau _\text {w}/\rho }$ which again relies on the shear stress at the wall $\tau _\text {w} = \mu du/dx$. The graphs reveal a peak in the interval of dimensionless times between 5 and 10. The peak is held for a small time before the Reynolds number decreases.

### Wall-normal statistics

The quantities that characterize the effects of turbulence such as the kinetic energy *k* and the coherent structure function $F_{\text {CS}}$ are assessed. The coherent structure function is defined as:

4 $$\begin{aligned} F_\text {CS} = \frac{Q}{E} \end{aligned}$$

which is the ratio of the second invariant of the velocity gradient tensor:

5 $$\begin{aligned} Q = -\frac{1}{2}\frac{\partial {u}_\text {j}}{\partial x_\text {i}}\frac{\partial {u}_\text {i}}{\partial x_\text {j}} \end{aligned}$$

and its respective magnitude:

6 $$\begin{aligned} E = \frac{1}{2}\frac{\partial {u}_\text {j}}{\partial x_\text {i}}\frac{\partial {u}_\text {j}}{\partial x_\text {i}} \end{aligned}$$

The coherent structure function is an instructive quantity in the context of this work because it measures the relation between dissipation and enstrophy, i.e. two fundamental quantities to characterize the structure of turbulent flows. Being a strictly bounded quantity ($-1< F_{\text {CS}} < +1$), it is also mathematically elegant and the iso-contour $F_{\text {CS}} = 0$ identifies the dissipation-enstrophy equilibrium. This allows to infer the size of coherent, i.e. connected, flow structures in an instantaneous sense and the dominance of dissipation or enstrophy in an averaged sense. It is worth mentioning that $F_{\text {CS}}$ can be seen as the suitably normalized Q-criterion which is commonly used in the literature to identify enstrophy-dominated flow regions in turbulent flows. Averaging was performed for the wall-parallel slices and the averages are plotted versus the wall-normal distance. The profiles are evaluated in dimensionless time intervals of 2.5 for all used grid-resolutions. This form of presentation confirms that the grids perform similar even for the near wall region. In the following, it can be observed by the markers that the finest grid leads to a minimum of two grid points within the first wall unit at dimensionless times of 5.0 and 7.5.

**Figure 4:**
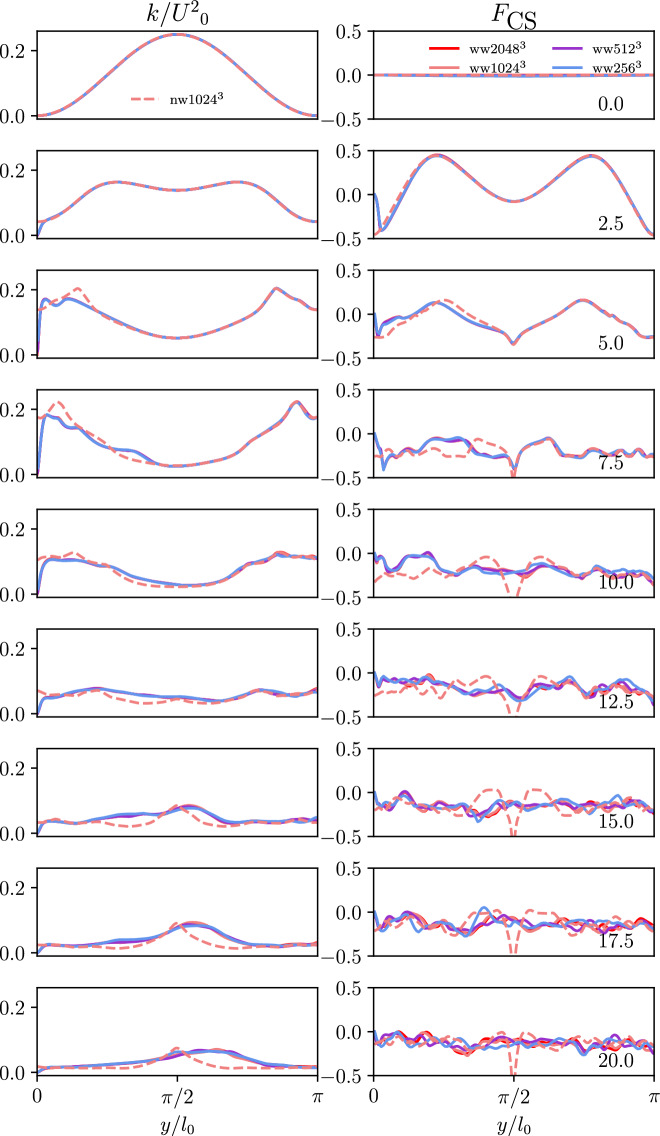
Time evolution of the wall-normal profiles *y* of the kinetic energy *k* and the coherent structure function $F_{\text {CS}}$ for all grid-resolutions of the TGV and WTGV. For the WTGV the wall is located at $y = 0$ and the domain center corresponds to $y = \pi$.

Figure 4 shows the averaged profiles of the kinetic energy *k* and the coherent structure function $F_{\text {CS}}$ in normalized units from the wall to the domain center for the TGV and WTGV. Only the finest grid of the TGV is included in these results, as the resolution of the TGV is already well studied in literature. No difference can be observed for the kinetic energy between the different cases at the first timestep due to the identical initialization. Until time 7.5 all curves remain identical for the channel center despite the WTGV and TGV curves separating due to the influence of the wall progressively starting to affect the flow for larger values of *y*. The kinetic energy at the wall is forced to zero due to the no-slip condition, while for the TGV the energy rises at the periodical boundary. First small differences between the different resolutions of the WTGV can be found from dimensionless times of 10.0 onwards. Opposed to the differences between TGV and WTGV, the differences between the WTGV resolutions propagate from the channel center towards the wall. The formation of the peak kinetic energy of the TGV until dimensionless times of 20.0 can also be found for the WTGV although being shifted to slightly higher values of *y*. The symmetry of the TGV profile remains. Most similarities between the TGV and WTGV have vanished at dimensionless times of 20.0. Up to this point the resolutions of $1024^3$ and $2048^3$ for the WTGV show only little deviation and hence the results can be considered converged. The coherent structure function is displayed in the second column. The coherent structure function remains zero in the first timestep due to the strain-rotation equilibrium given by the initialization. For the WTGV the coherent structure function holds values of zero at the boundaries due to the no-slip walls. As observed for the two previous quantities, the WTGV results on different grids remain identical until dimensionless times of 7.5. The TGV profile shows deviations near the wall. Initially two peaks of rotation dominated flow can be found, which then start to flatten out. Overall the values of the coherent structure function remain negative due to dominance of strain in the flow. The coherent structure function settles at values of around $-0.1$ for dimensionless times of 10.0 onwards which also is the value found by Kobayashi in the case of homogeneous isotropic turbulence^19^. In fact it can also be observed that the WTGV approaches this state faster than the TGV as the latter features a notable peak at these simulation times. A distinct peak starts to form for the TGV case at this point which becomes even more sharp with the simulation proceeding while the WTGV profile remains comparably homogeneous. Thus, it can be concluded that away from the wall the state of homogeneous isotropic turbulence is reached faster for the WTGV than for the TGV.

**Figure 5:**
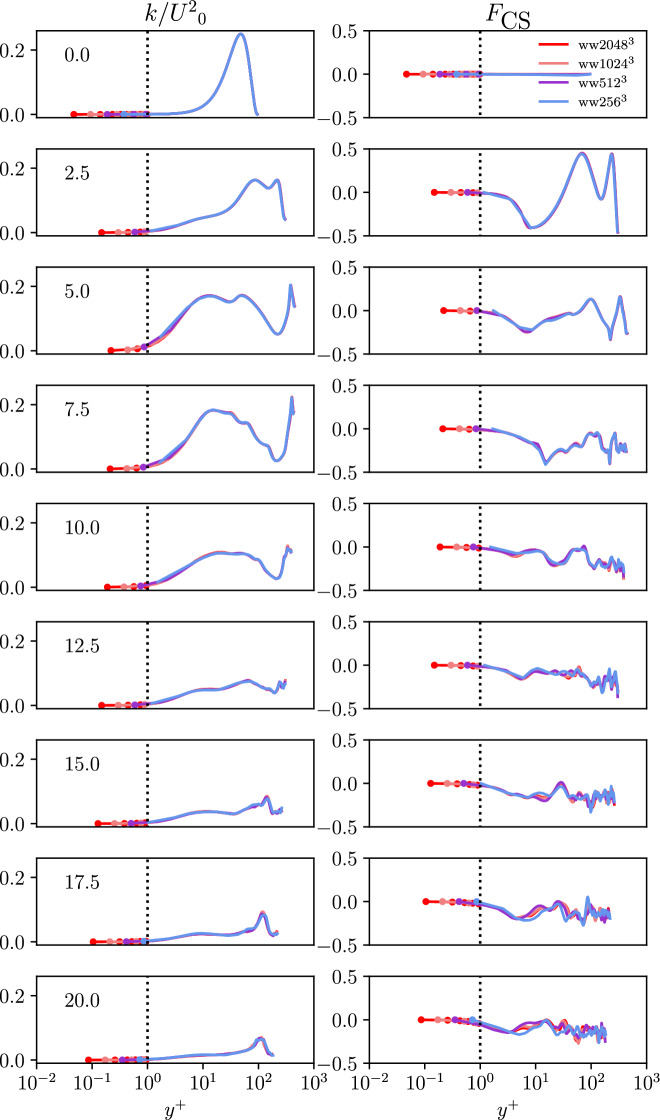
Time evolution of the wall-normal profiles in viscous wall units $y^+$ of the kinetic energy *k* and coherent structure function $F_{\text {CS}}$ for all grid-resolutions of the WTGV in a semi-logarithmic diagram. For the WTGV the wall is located at $y =0$.

In Fig. 5, the averaged profiles of the kinetic energy *k* and the coherent structure function $F_{\text {CS}}$ can be seen in a similar manner as in Fig. 4, however being plotted versus the distance from the wall in viscous wall units $y^+$ to allow for a more thorough analysis of the near-wall behavior.

The temporal evolution of the near-wall stresses will be presented to further investigate the suitability for wall modeling of this case. A popular choice for the assessment of models and methods near walls is the turbulent channel flow as shown by Moser et al.^20^ and Hoyas et al.^21^. In the case of the turbulent channel flow the strongest turbulent activity can be found in the buffer layer region. The wall scaling of the Reynolds stresses in channel flows is well known and forms the fundament to evaluate and calibrate the behaviour of turbulence models in the vicinity of walls. To assess the effect of the wall in the channel flow and in the WTGV the scaling of the stresses in the viscous sublayer is compared. Taylor series expansions of the velocity profiles at the wall can be written and evaluated using the no-slip boundary condition at the wall and the concept of the two-component flow combined with the continuity equation^22^. Scaling laws can be formulated using the lowest order terms of the series expansion as they dictate the asymptotic behavior near the wall:

7 $$\begin{aligned} \begin{aligned} \langle u^{\prime }u^{\prime }\rangle&= {\fancyscript{O}}\left( y^2\right) \\ \langle v^{\prime }v^{\prime }\rangle&= {\fancyscript {O}}\left( y^4\right) \\ \langle w^{\prime }w^{\prime }\rangle&= {\fancyscript {O}}\left( y^2\right) \\ \end{aligned} \end{aligned}$$

with the stress tensor being symmetric. It has to be recalled that the wall-normal velocity component in this work is *v*.

**Figure 6:**
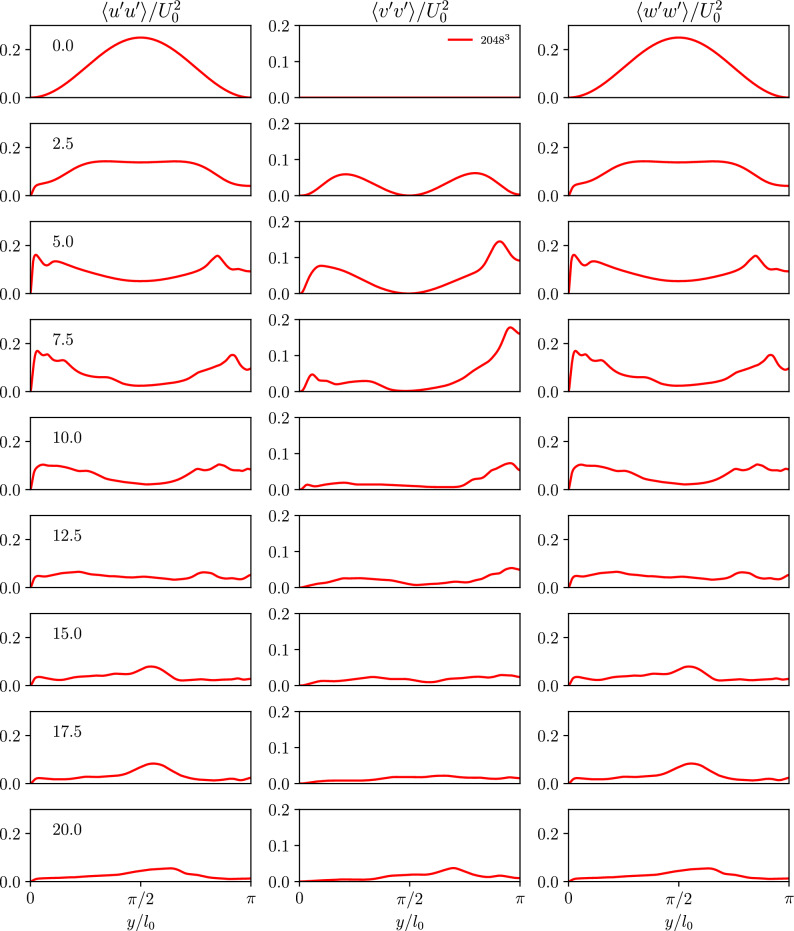
Time evolution of the wall-normal profiles *y* of the normalized stress components $\langle u^{\prime }u^{\prime }\rangle /U_0^2$, $\langle v^{\prime }v^{\prime }\rangle /U_0^2$ and $\langle w^{\prime }w^{\prime }\rangle /U_0^2$ for the finest grid-resolution of the WTGV. The wall is located at $y = 0$ and the domain center corresponds to $y = \pi$.

**Figure 7:**
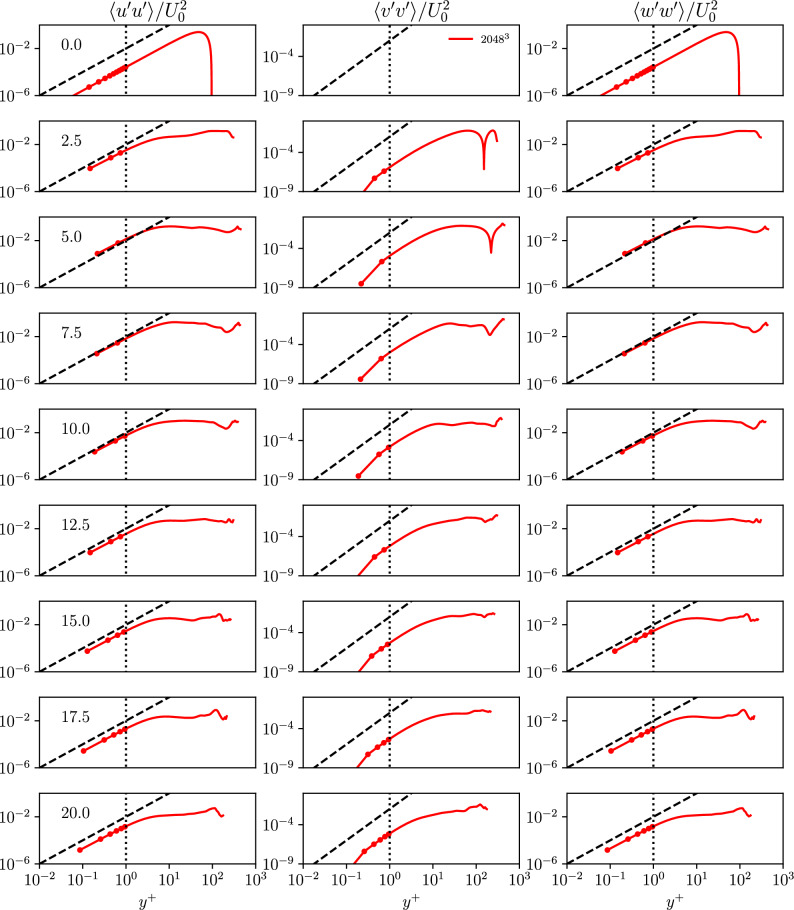
Time evolution of the near wall profiles in viscous wall units $y^+$ of the normalized stress components $\langle u^{\prime }u^{\prime }\rangle /U_0^2$, $\langle v^{\prime }v^{\prime }\rangle /U_0^2$ and $\langle w^{\prime }w^{\prime }\rangle /U_0^2$ for the finest grid-resolution of the WTGV in a double-logarithmic diagram.

The temporal evolution of the first three computed normalized stresses from the WTGV within the viscous sublayer are shown in Fig. 6. The scaling behavior is added to each stress component to allow for easier comparison in Fig. 7. The scaling of the wall-parallel Reynolds stress components $\langle u^{\prime }u^{\prime }\rangle$ and $\langle w^{\prime }w^{\prime }\rangle$ agrees well with the expected behaviour. However, in contrast to the channel flow the $\langle v^{\prime }v^{\prime }\rangle$ stress shows a steeper slope close to the wall. This is nevertheless not in contradiction with Eq. 7 which states that the $\langle v^{\prime }v^{\prime }\rangle$ stress changes at least with the wall-normal distance to the power of four. In addition, all off-diagonal components of the Reynolds stress tensor are zero in the WTGV configuration (again no contradiction with Eq. 7).

The aforementioned observations imply that the instationary nature of the boundary layer featured within the proposed WTGV case behaves differently from the stationary boundary layer in the turbulent channel flow. Hence, the instationary boundary layer in this case forms a new aspect which is considered valuable in the study of turbulence and wall modeling.

The walls clearly affect the dynamics of the (standard) TGV configuration but at the same time the symmetry conditions and the unsteadiness of the flow distinguish the WTGV clearly from other wall bounded shear flows like the channel flow configuration. This can be conveniently characterized with the help of the Lumley triangle^24^. Its boundaries are given as a function of the second invariant:

8 $$\begin{aligned} \text {II}\left( a\right) = \left( \text {trace}\left( a\right) ^2-\text {trace}\left( a^2\right) \right) /2 \end{aligned}$$

and third invariant:

9 $$\begin{aligned} \text {III}\left( a\right) = \left( \text {det}\left( a\right) \right) \end{aligned}$$

of the anisotropy tensor:

10 $$\begin{aligned} a_\text {ij} = \langle u^\prime _\text {i} u^\prime _\text {j}\rangle / \langle u^\prime _\text {i} u^\prime _\text {i}\rangle -\delta _\text {ij}/3 \end{aligned}$$

and by introducing the variables:

11 $$\begin{aligned} \eta = \left( -\frac{1}{3}\text {II}\left( a\right) \right) ^{1/2} \end{aligned}$$

and:

12 $$\begin{aligned} \xi = \left( -\frac{1}{2}\text {III}\left( a\right) \right) ^{1/3} \end{aligned}$$

**Figure 8:**
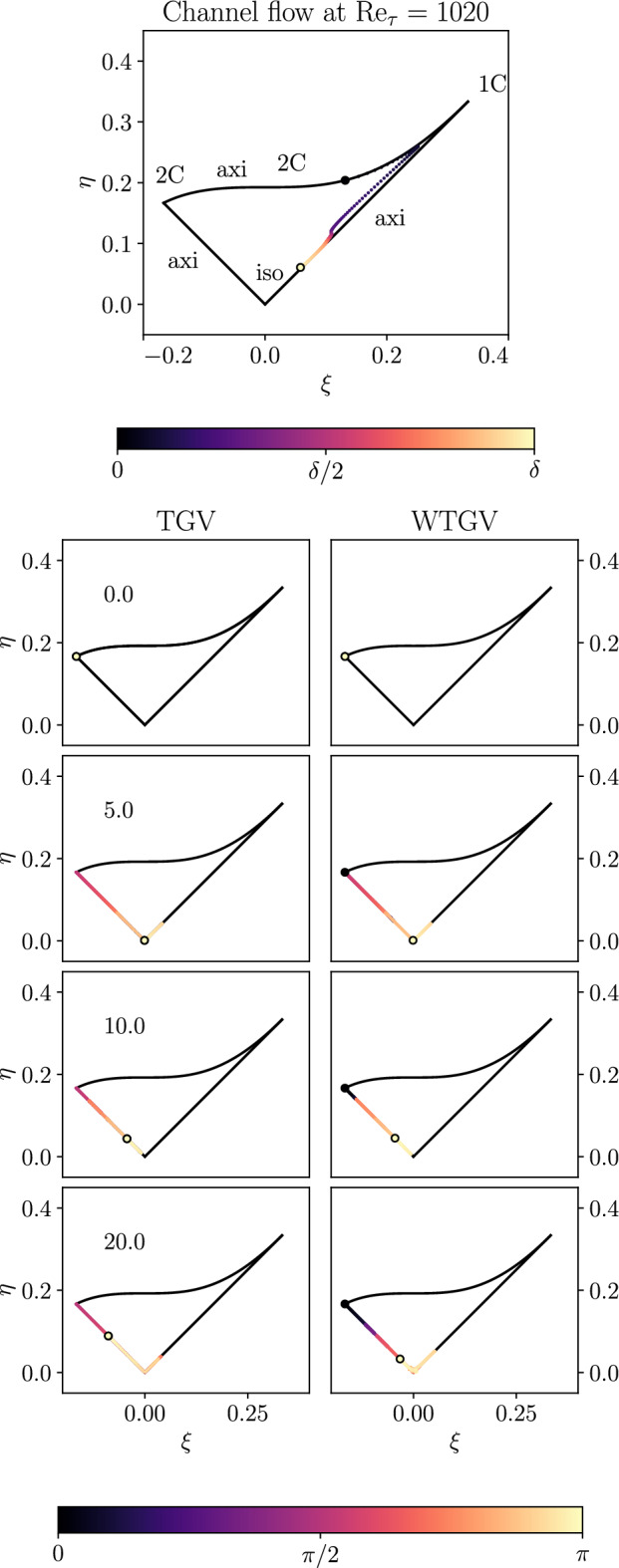
Anisotropy of the Reynolds stresses in the Lumley triangle. The color coding represents the wall-normal distance with the black and white circle indicating the wall and the domain center. The top figure shows a channel flow for Re$_\tau =1020$ by Abe et al.^23^. The main panel shows the TGV (left) and the WTGV (right) for four different instants in time $t = 0, 5, 10, 20$.

Any physically realizable state of the anisotropy tensors $a_\text {ij}$ lies within the triangles shown in Fig. 8. The left and right borders of the triangle represent an axisymmetric contraction and expansion respectively, while the upper boarder denotes the two-component state. Finally, the isotropic state corresponds to the lower corner $\left( \xi ,\eta \right) =\left( 0,0\right)$. In order to contrast the turbulence state in the WTGV with equilibrium wall bounded flows the behavior of a channel flow by Abe et al.^23^ is shown in the top part of Fig. 8. Very close to the wall, in the viscous sublayer of the channel flow, the turbulence is essentially of two-components, with *v* being much smaller than *u* and *w*. Anisotropy reaches a peak at a dimensionless wall distance of about $y^+ \approx 7$ close to the 1C state and subsequently becomes increasingly isotropic towards the channel center. The main panel of Fig. 8 shows the Lumley triangles for the TGV (left) and the WTGV (right) for four instants in time. The Reynolds stress components are averaged in planes parallel to the wall and the color coding represents the wall distance with the black and white circle denoting $y=0$ and $y=\pi$ respectively. For $t=0$ the *v*-velocity component is zero and the fluctuating *u* and *w* velocity components have equal strength, such that the flow state is concentrated in a single point in the axisymmetric two-component corner. With increasing time the turbulent states populate the complete left border (i.e. the so called axisymmetric contraction) and to a small extent the right axisymmetric expansion branch for small values of $\xi$. It is worth noting that the left border of the triangle can be associated with the phenomenon of vortex stretching^25^. For the standard TGV case the *y*-direction is periodic and the flow is symmetric with respect to the plane $y=\pi$. Therefore, for all instants in time the black and white circle coincide with each other in case of the TGV. By contrast for the WTGV case the turbulent state remains mostly two-component axisymmetric in the vicinity of the wall while towards the center of the WTGV the flow becomes increasingly isotropic. This behavior is clearly different to equilibrium wall bounded flows with a mean flow and it will be interesting to test and develop turbulence models for this new benchmark configuration featuring unsteady wall bounded flows.

### Scalar transport

To achieve a case design of the WTGV allowing for scalar studies, a passive scalar was included into the setup. The scalar is transported from the wall into the fluid. The wall is set to hold scalar values of unity, e.g. representing a fixed temperature boundary, and the fluid holds values of zero at the time of initialization.

**Figure 9:**
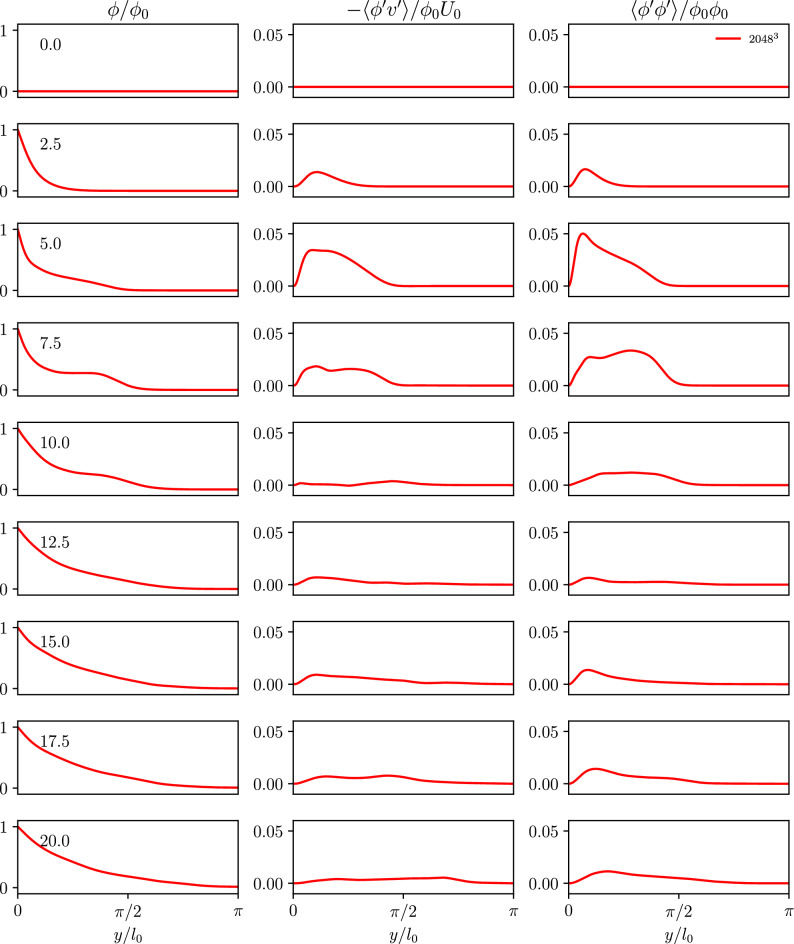
Time evolution of the wall-normal profiles *y* of the passive scalar $\phi$, the scalar flux $-\langle \phi ^{\prime }v^{\prime } \rangle /\phi _0 U_0$ and its variance $\langle \phi ^{\prime }\phi ^{\prime } \rangle /\phi _0 \phi _0$ for the WTGV. The wall is located at $y = 0$ and the domain center corresponds to $y = \pi$.

To assess the temporal distribution of the passive scalar in the domain, wall-parallel averaging was performed in the same manner as in Figs. 4 and 5. Figure 9 shows the temporal evolution of the wall-parallel averages of the passive scalar in the WTGV for the highest resolution. Due to the Schmidt number significantly below unity the profiles for all resolutions are well converged and thus, only the finest grid is shown. Initially the domain holds scalar values of zero and the scalar can be seen to propagate into the domain according to the fixed value boundary condition. The curves show regressive behavior for all times. Slight deformation of the profiles can be seen in the interval of dimensionless times from 5.0 to 10.0 when the vortex break-down is in full progress. The curves show a plateau within the first quarter of the domain which is vanishing with the simulation proceeding. Overall the scalar values show smaller changes towards the end of the simulation.

**Figure 10:**
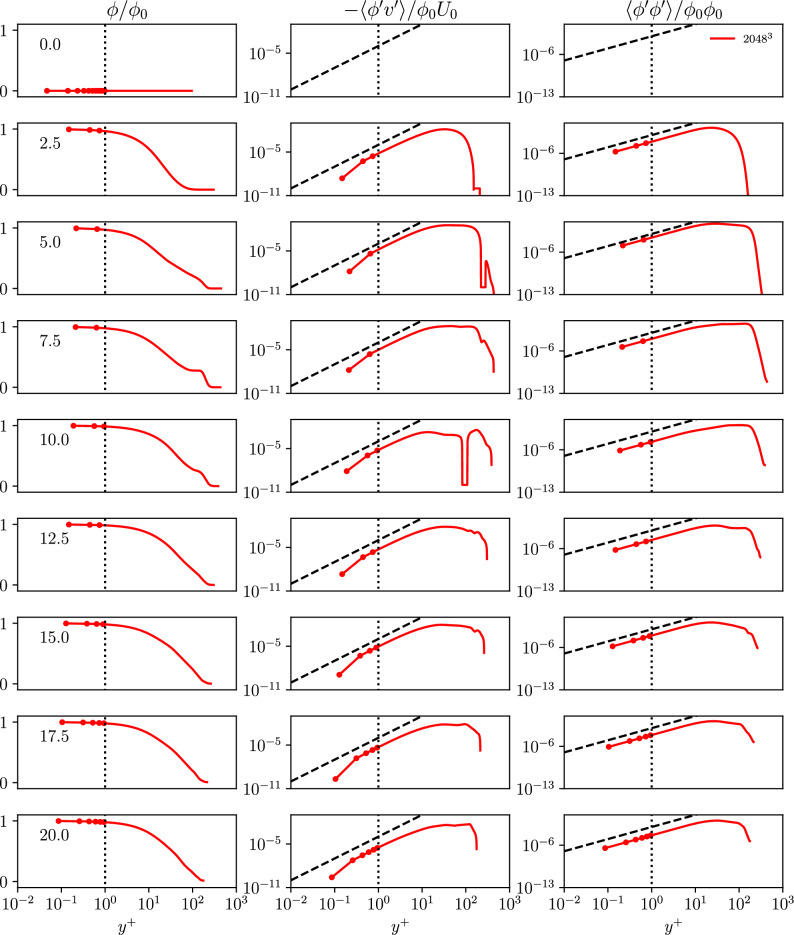
Time evolution of the wall-normal profiles in viscous wall units $y^+$ of the passive scalar $\phi /\phi _0$ in a semi-logarithmic diagram, as well as the scalar flux $-\langle \phi ^{\prime }v^{\prime }\rangle /\phi _0 U_0$ and its variance $\langle \phi ^{\prime }\phi ^{\prime }\rangle /\phi _0 \phi _0$ for the WTGV in a double-logarithmic diagram.

To allow for a better insight into the near wall region the plot is shown using viscous wall units in Fig. 10. The visualization using viscous wall units shows the plateau in a more pronounced manner and reveals that remainders of the plateau in fact can be observed till the end of the simulation.

The correlation of the scalar and velocity fluctuations is analyzed with respect to the near wall behavior. Similar as for the stresses, scaling laws for the near wall behavior of the correlated quantities can be obtained^22^:

13 $$\begin{aligned} \begin{aligned} \langle \phi ^{\prime }\phi ^{\prime } \rangle&= {\fancyscript {O}}\left( y^2\right) \\ \langle \phi ^{\prime }v^{\prime } \rangle&= {\fancyscript {O}}\left( y^3\right) \\ \end{aligned} \end{aligned}$$

Because of the periodic boundary conditions, there can be no wall-parallel turbulent scalar flux. The profile of the wall-normal flux component $\langle \phi ^{\prime }v^{\prime } \rangle$ has a slightly higher slope than predicted by Eq. 13, which is consistent with the observed scaling for $\langle v^{\prime }v^{\prime } \rangle$. The scalar variance $\langle \phi ^{\prime }\phi ^{\prime } \rangle$ agrees well with the slope predicted by Eq. 13. The observed behaviour for the scalar flux implies that for the WTGV, the alignment given by the gradient flux approximation would be correct, and modeling of the averaged flux will be limited to the determination of the appropriate turbulent diffusivity.

## Summary

A new benchmark case for the study of DNS and LES models has been proposed and investigated. The case is based on the known Taylor–Green vortex but the periodic conditions at the boundaries of the domain, in the direction where the initial velocity is at rest, are replaced with a no slip wall. The absence of mean velocity in the configuration is deliberately chosen to represent a non-standard boundary layer flow. It is worth noting that various boundary layer flows, such as the oscillatory boundary layer or the flow in an combustion engine without pronounced tumble or swirl motion, can exhibit a lack of mean velocity. The primary objective of presenting this particular test case is to challenge existing wall models and make valuable contributions towards the development of more comprehensive formulations capable of accommodating a wide range of scenarios. These formulations would encompass cases where oscillation or turbulent fluctuation surpasses the mean velocity, as well as the more familiar scenario with a notable mean velocity. Furthermore, it is proposed that introducing a mean velocity in the flow’s wall parallel direction, similar to a channel flow setup, is indeed feasible. However, it should be noted that such an addition would introduce additional complexity, primarily due to the presence of another transient component, which is beyond the scope of this current study. This aspect serves as a potential avenue for future research and investigation.

The WTGV differs from existing wall bounded flow benchmarks by the absence of a mean flow and by its global unsteadiness. As a consequence, the wall scaling of the Reynolds stresses and the flow anisotropy are different to steady state boundary layer flows. Hence, due to its simplicity, this configuration represents a potentially useful benchmark to challenge existing turbulence and wall models.

## Data Availability

The datasets used and/or analysed during the current study available from the corresponding author on reasonable request.
